# Gastroprotective Effects of the Aqueous Extract of Finger Citron Pickled Products against Ethanol-Induced Gastric Damage: In Vitro and In Vivo Studies

**DOI:** 10.3390/foods12122355

**Published:** 2023-06-13

**Authors:** Xiaoai Chen, Dan Yang, Qun Wang, Aimei Zhou

**Affiliations:** Guangdong Provincial Key Laboratory of Nutraceuticals and Functional Foods, College of Food Science, South China Agricultural University, Guangzhou 510642, China; cxarky@126.com (X.C.); yangdan242424@163.com (D.Y.); 249885990@scan.edu.cn (Q.W.)

**Keywords:** finger citron pickled products, gastric mucosa, ethanol injury, GES-1 cell, gastric ulcer rat, protection mechanism

## Abstract

Finger citron pickled products (FCPP), as folk remedies, are famous in southern China for protecting gastric mucosa. However, the gastric mucosa protection of FCPP has not been reported yet, and its effective mechanism is unclear. In this study, the protective mechanism of FCPP aqueous extract on gastric mucosa was investigated in vitro and in vivo for the first time, using human gastric mucosa epithelial cells (GES-1) and acute alcoholic gastric ulcer rat model respectively. Furthermore, we also investigated the main substances in the aqueous extract that exert gastroprotective activity using a GES-1 scratch test and basic chemical composition analysis. FCPP aqueous extract was found to play a protective and reparative role in GES-1 by promoting the secretion of trefoil factor thyroid transcription factor 2 (TFF2) and inhibiting the secretion of tumor necrosis factor-*α* (TNF-*α*) in cells damaged by alcohol. The ulcer index of gastric tissue induced by alcohol was significantly decreased (*p* < 0.01) after pretreatment with FCPP aqueous extract, indicating that FCPP aqueous extract had a good protective effect on the stomach mucosa. Moreover, FCPP aqueous extract could increase superoxide dismutase (SOD) activity and inhibit malondialdehyde (MDA) content, exhibiting good antioxidant capacity. Aqueous extract of FCPP could also effectively inhibit the increase of cytokines TNF-*α*, interleukin-1*β* (IL-1*β*) and interleukin-6 (IL-6) in serum of rats, and promote the increase of anti-inflammatory cytokines interleukin-10 (IL-10) to some extent. Furthermore, FCPP aqueous extract could inhibit the expression of nuclear factor kappa-B (NF-*κ*B/P65) protein, caspase-1 protein and IL-1*β* protein in the gastric tissue of rats, while promoting the expression of I*κ*B*α* protein, indicating that the gastric mucosa protection effects of FCPP aqueous extract were mainly dependent on the NF-κB/caspase-1/IL-1*β* axis. The polysaccharides in FCPP aqueous extract might be the main components that exerted gastroprotective activity, as demonstrated by GES-1 cell scratch assay. This study confirmed that FCPP aqueous extract presented promising potential in protecting gastric mucosa and avoiding gastric ulcers, which could provide an experimental basis for further utilizing the medicinal value and developing new products of FCPP.

## 1. Introduction

Gastric ulcer disease is one of the most common types of stomach disease, and the worsening of severe gastric ulcers may further induce gastric cancer [1,2]. Gastric ulcers are mainly caused by *Helicobacter pylori* infection, dietary stimulation, some bad living habits like drinking and smoking and other factors that damage gastric mucosa [3]. The integrity of gastric mucosal function plays a decisive role in the health of the stomach, which depends on the balance between its resistance and damage level, so mild gastric ulcers can heal without treatment [4]. However, with the accelerated pace of life, drinking has become a way to relieve stress for many people, so the number of patients with alcoholic damage to the gastric mucosa is also increasing. Related studies have shown that drinking can lead to changes in the protective factors in gastric mucosa, and then cause a series of inflammatory reactions [5,6]. Excessive drinking can produce toxic effects, stimulate gastric acid secretion, damage gastric mucosa and aggravate ulcer symptoms [7]. Inflammation and oxidative stress are involved in the pathogenesis of gastric ulcers. The NF-*κ*B/NLRP3 inflamma some pathway is an important pathway that regulates the expression and secretion of inflammatory factors [8]. Studies have found that the NF-*κ*B/NLRP3 inflamma some pathway can be divided into three steps. Firstly, exogenous attack factors enter the body and stimulate the decomposition of the inhibitor of the NF-*κ*B (I*κ*B) complex, resulting in the phosphorylation of free I*κ*B protein, which further activates the phosphorylation of nuclear factor kappa-B (NF-*κ*B/P65) into the nucleus for reverse transcription, and up-regulates the expression of tumor necrosis factor-*α* (TNF-*α*), interleukin-1*β* (IL-1*β*), interleukin-6 (IL-6) and interleukin-18 (IL-18) genes [9]. Secondly, macrophages are stimulated to activate NLRP3 protein expression regulated by NF-*κ*B, which further promotes the apoptosis-associated speck-like protein (ASC) and caspase-1 inflammatory complex. Finally, decomposition of the ASC-Caspase-1 inflammatory complex leads to the activation of free caspase-1, which further induces the secretion of cytokines IL-1*β* and IL-18 [10]. Moreover, lipid peroxidation is an oxidation-induced stress response and an important pathogenesis of gastric mucosal injury [11]. Superoxide dismutase (SOD) activity and malondialdehyde (MDA) levels are potential important indicators of antioxidant capacity in vivo, which can reflect the rate and intensity of lipid peroxidation and indirectly reflect the degree of tissue peroxidation damage [12,13].

Finger citron pickled products (FCPP), known as “Lao Xiang Huang”, are one of the rare traditional Chinese medicinal materials in the south of China. FCPP is produced from the rutaceous plant finger citron (*Citrus medica* L. var. sarcodactylis Swingle) by pickling and fermentation. Fresh finger citron is perishable and is too spicy and bitter for direct consumption; therefore, it is usually produced in pickled products to prolong its storage time and ameliorate its taste [14]. The pickling process of finger citron is very complex, consisting of many steps, including salting, desalting, sugaring, cooking and drying [15]. After drying, finger citron was sealed in the jar for long-term fermentation. During the pickling and fermentation process, the color, shape, flavor and medicinal value—such as the gastric protective effect of finger citron—are significantly changed, especially the latter two [16]. Villagers in southern China often use FCPP to treat stomachache, abdominal distension, vomiting, hiccups, phlegm, cough, asthma and other diseases [17]. In addition, the longer the fermentation time of FCPP, the better the medicinal effect. Although FCPP has been widely accepted as an effective folk means to protecting gastric mucosa, the scientific evidence of FCPP in protecting the stomach has not been reported yet, and its protective mechanism is still unclear, which hinders the rapid development of the finger citron industry and is not good for the development of various deep-processed products of FCPP. In our previous experiment, a GES-1 cell model was used as an evaluation method to compare the protective effects of different solvent extracts on gastric mucosa, and the results showed that the optimal activity group was water extract. Therefore, human gastric mucosal epithelial cells (GES-1) were used in this study to investigate the preventive and protective effects of FCPP aqueous extract on gastric mucosal injury. Moreover, the relevant protective mechanism was further investigated with rat acute alcoholic gastric ulcer model. Finally, GES-1 cells were used to infer the main substances that exert activity in FCPP aqueous extracts. This study is of great significance for the development of new FCPP products and to promote the development of the FCPP deep processing industry.

## 2. Materials and Methods

### 2.1. Materials and Chemicals

FCPP (fermented for 3 years) was provided by Guangdong Zhancui Food Co., Ltd. (Guangzhou, China) and stored at −20 °C. GES-1 was supplied by Shanghai cell bank, Chinese Academy of Sciences (Shanghai, China). The healthy SPF male SD rats were provided by Guangdong Medical Laboratory Animal Center (Guangzhou, China) with the laboratory animal certification number of No. 44007200085002 and the license number of SCXK2018-0002.

Enzyme linked immunosorbent assay (ELISA) kits, including thyroid transcription factor 2 (TFF2, Cat.No.CSB-EL023432HU), was bought from Cusabio Biotech Co., Ltd (Wuhan, China), TNF-*α* (Cat.No.EHC103a), IL-6 (Cat.No.ERC003), IL-10 (Cat.No.ERC004) and IL-1*β* (Cat.No.ERC007)were bought from NeoBioscience Biological Technology Co., Ltd. (Beijing, China). SOD (Cat.No.A001-1-2) and MDA (Cat.No.A003-1-2)assay kitswere purchased from the Nanjing Jiancheng Bioengineering Institute (Nanjing, China). NF-*κ*B (P-65, Cat.No.66535-1-Ig), I*κ*B*α* (Cat.No.66418-1-Ig), caspase-1 (Cat.No.22915-1-AP), IL-1*β* (Cat.No.16806-1-AP) and *β*-actin (Cat.No.66009-1-Ig) were purchased from Proteintech (Chicago, CA, USA). All other reagents used in this study were analytic grade, unless otherwise specified.

### 2.2. FCPP Aqueous Extract Preparation

According to the polarity, petroleum ether, n-butanol, ethyl acetate, ethanol, methanol and aqueous were used to extract FCPP step by step. A total of 10 g of dry powder was mixed with an appropriate amount of distilled water to obtain a muddy water suspension, which was shaken and transferred to the separating funnel. After petroleum ether was added into the separating funnel, it was fully shaken and allowed to stand until the solution was completely layered. The petroleum ether extract was then released from the upper layer, and this process was repeated until the petroleum ether layer became transparent and colorless. This method was repeated, and solvents such as n-butanol, ethyl acetate, ethanol and methanol were sequentially extracted step by step. After extraction, each solution was left to stratify, all organic solvents were removed by rotary evaporation and the aqueous solution was finally separated as the aqueous layer extract. The concentrated solution was repeatedly freeze-dried to obtain the solids of FCPP aqueous extract, which were then put into the drying vessel.

### 2.3. Effect of FCPP Aqueous Extract on GES-1 Cell Alcohol Injury

The ethanol damage model was performed following the method described by Zhu et al., with some modifications [18]. Cell lines in the logarithmic growth phase were digested, suspended and inoculated on 12-well culture plates (4 × 10^5^ cell/well). The cells were divided into a normal control group, an ethanol group, an FCPP aqueous extract group and a teprenone group (80 μM). The FCPP aqueous extract and teprenone were diluted to a fixed concentration with complete medium. Each group had three duplicate wells, which were cultivated at 37 °C and 5% CO_2_ for 24 h. Ethanol was added to each well to reach the final concentration of 0.8 M, except for the normal control group. After 4 h of stimulation, the supernatant was removed, and PBS was added to wash each well. The complete medium containing FCPP aqueous extract was then added to each FCPP aqueous extract group, while the fresh complete medium containing 10% fetal bovine serum was given to the normal control and ethanol groups. All groups were cultured at 37 °C for 24 h in a carbon dioxide (5%) incubator, and the cell fluid was collected. After centrifugation at 2000 rpm for 10 min, the supernatant was separated and TFF2 and TNF-*α* levels were determined.

### 2.4. Animal Treatment

Male SD rats (200 ± 20 g) were fed adaptively for 7 days in an SPF animal laboratory with a feeding temperature of 23~26 °C, a relative humidity of 40~70% and intermittent lighting during day and night. The animals were free to eat and drink during the experiment (which lasted for 3 weeks). Sixty rats were randomly divided into six groups of 10 each, as shown in Table 1: normal group; model group; and omeprazole group (50 mg·kg^−1^·d^−1^, body wt., i. g); as well as high-dose (800 mg·kg^−1^·d^−1^, body wt., i. g); medium-dose (400 mg·kg^−1^·d^−1^, body wt., i. g); and low-dose (100 mg·kg^−1^·d^−1^, body wt., i. g) groups of FCPP aqueous extract. Rats were administered with drugs at 5 mL·kg^−1^·d^−1^, body wt., i. g and normal and model groups were administered with an equal volume of normal saline (i. g) for 15 consecutive days. The animals were checked and weighed daily during the administration. On the day of the last administration, rats in each group were fasted, but allowed to drink freely for 24 h. The normal group was administered with equal volume of normal saline the following day, and rats in other groups were administered with 5 mL·kg^−1^ (body wt., i. g) of anhydrous ethanol for modeling [19]. Forty minutes after modeling, the rats were anesthetized with chloral hydrate. The abdominal cavity of the rats was then opened, and blood was taken from the aorta with a blood collection needle. After the collection of blood, the anterior abdominal wall of the rats was cut, and the stomach was first taken out, followed by the liver, kidney, spleen and thymus. The gastric tissue of the rats was immediately cut along the great bend from the cardia at the same time, which was washed with normal saline, dried, flattened and photographed quickly. The experimental protocol was approved by the Experimental Animal Ethics Committee of South China Agricultural University, with approval number 2020b116.

### 2.5. Gastric Ulcer Evaluation

After taking photos of the gastric tissue, the area of the gastric ulcer and the total area of the glandular stomach were calculated by ImageJ image software. The ulcer index (UI) and ulcer inhibition rate (UIR) were calculated according to Formulas (1) and (2).

(1) $$\mathrm{UI}\;(\%)=\frac{\mathrm{UA}}{\mathrm{TA}}\;\times \;100\%$$

where UA and TA are the area of gastric ulcer and the total area of corresponding gastric tissue glands, respectively.

(2) $$\mathrm{UIR}\;(\%)=100\%-\frac{\mathrm{DUI}}{\mathrm{MUI}}\;\times \;100\%$$

where DUI and MUI represent the gastric ulcer index in the drug group and the model group, respectively.

### 2.6. Histopathological Analysis

After macroscopic observation, part of the gastric tissue was immersed in 10% formalin reagent, then dehydrated and embedded in paraffin. Histopathological analysis was performed under a light microscope following hematoxylin-eosin staining (H&E) and Periodic acid–Schiff staining (PAS).

### 2.7. Determination of Serum Levels

The blood collected from each rat (see Section 2.4) was centrifuged for 10 min at 3000 r/min to separate the serum for the measurement of TNF-*α*, IL-1*β*, IL-6 and IL-10 using ELISA kits, together with the determination of SOD and MDA using assay kits.

### 2.8. Western Blot Analysis

The expression levels of the related proteins in the NF-*κ*B and NLRP3 pathways, including NF-*κ*B (the dilution ratio is 1:2000), I*κ*B*α* (the dilution ratio is 1:7000), caspase-1 (the dilution ratio is 1:1000) and IL-1*β* (the dilution ratio is 1:750) in gastric tissues, were investigated by western blot analysis, according the method described by Nabil et al. [20].

Firstly, a radio imunoprecipitation assay (RIPA) lysis buffer was used to extract total protein from gastric tissue. Following that, lysates were subjected to 10% SDS-PAGE and then transferred to nitrocellulose NC membranes. After a 1 h blocking with 5% (*w*/*v*) nonfat milk in phosphate buffer solution containing tween 20 (PBST), the NC membranes were incubated overnight at 4 °C with anti-NF-*κ*B, anti-I*κ*B*α*,anti-IL-1*β* and anti-caspase-1andanti-*β*-actin monoclonal antibodies. Afterwards, they were incubated with the indicated antibodies overnight at 4 °C. Then, the membranes were subsequently washed with PBST and incubated for 1 h at room temperature, with the appropriate secondary anti-bodies conjugated with horseradish. The immunoreactive band was detected using the SuperECL Plus kit (Advansta Co., Menlo Park, CA, USA) and *β*-actin was used as internal control. Imaging and densitometry analyses were performed using an imaging system and Quantity One analyzer software (Epson, Suwa, Nagano, Japan & Bio-Rad, Hercules, CA, USA).

### 2.9. Cell Scratch Assay and Chemical Composition Analysis

To explore the main substances that contributed to the gastroprotective effects in FCPP aqueous extract, the extract was subjected to precipitation with 80% (*w*/*v*) ethanol, followed by centrifugation at 4500 rpm for 15 min, after which the supernatant and the precipitate were collected. The precipitate was further deproteinized according to the method of Zhou [21]. Next, the supernatant and the precipitate were lyophilized and performed GES-1 cell scratch assay, respectively. Briefly, GES-1 cells were seeded in a 24-well plate at 2 × 10^5^ cells per well and grew to reach 95% confluence. After the cells were closely connected in a single layer, the cells were scratched vertically in the center of the 24-well plate with the head of a 200 μL gun. The floating cells were then washed with PBS solution, and the remaining cells in the 24-well plate were treated with ethanol precipitate (deproteined) and supernatant (100 µg/mL in DMEM), followed by cultivation at 37 °C and 5% CO_2_ for 24 h. Scratches in different groups were visualized and photographed before stimulation (0 h) and after 24 h under an inverted microscope. Cell migration ability was measured using ImageJ Pro Plus 6.0 software [12].

The ethanol precipitate (deproteined) of the FCPP aqueous extract were further subjected to chemical composition analysis. The content of total sugar and protein was determined by phenol-sulfuric acid colorimetric method and the Coomassie Brilliant Blue method, respectively, while the content of uronic acid was detected by the m-hydroxy diphenyl method [22,23,24]. Additionally, the Folin–Ciocalteau method was used to analyze the content of total phenolics [25].

### 2.10. Statistical Analysis

Each experiment was performed at least three times, and all values were represented as mean ± standard deviation (SD). Statistical analysis was carried out using SPSS 23.0 software (SPSS Inc., Chicago, CA, USA). The data was checked for normality and homogeneity, then a one-way analysis of variance (ANOVA) was adopted to compare the significant differences among all groups using Tukey’s HSD analysis. The differences were considered to be significantat *p* < 0.05.

## 3. Results

### 3.1. Effects of FCPP Aqueous Extract on Alcohol Damage of GES-1 Cells

**Figure 1:**
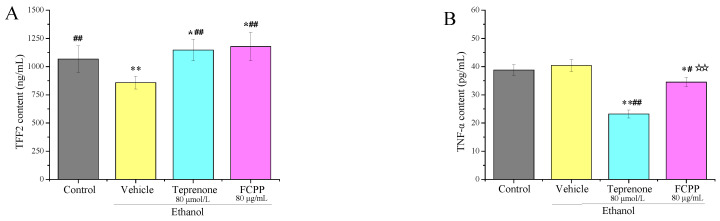
(**A**) Effects of FCPP aqueous extract on the secretion of TFF2 of GES-1 cells damaged by alcohol. (**B**) Effects of FCPP aqueous extract on the secretion of TNF-*α* of GES-1 cells damaged by alcohol. Control: cultured with cell culture medium only, and other groups were stimulated with ethanol before adding different drugs. Vehicle: stimulated with ethanol followed by the culture with cell culture medium. Teprenone: stimulated with ethanol followed by the culture with cell culture medium containing 80 ug/mL teprenone. FCPP: stimulated with ethanol followed by the culture with cell culture medium containing 80 µmol/L FCPP aqueous extract. FCPP: finger citron pickled products. * *p* < 0.05, ** *p* < 0.01 versus the control group; ^#^ *p* < 0.05, ^##^ *p* < 0.01 versus the vehicle group; ^☆☆^ *p* < 0.01 the low, medium and high FCPP extract doses versus the teprenone group, *n* = 3.

In order to explore the reparative effect of FCPP aqueous extract on GES-1 cells after alcohol injury, the contents of TFF2 and TNF-*α* in cell supernatant were determined. As shown in Figure 1A, the mean level of TFF2 was 1066.9 ng/mL in the control group, 858.2 ng/mL in the vehicle group, 1177.7 ng/mL in the FCPP aqueous extract group and 1146.8 ng/mL in the teprenone group. The FCPP aqueous extract group was significantly higher in TFF2 content than that of the vehicle group (*p* < 0.05) and had no significant difference with the positive control group (i.e., the teprenone group) (*p* > 0.05). While the mean level of TNF-*α* was 38.8 pg/mL, 40.4 pg/mL, 34.5 pg/mL and 23.2 pg/mL in each group, respectively, as shown in Figure 1B, the FCPP aqueous extract group had significantly lower levels than that of the vehicle group (*p* < 0.05). These results demonstrate that FCPP aqueous extract could promote the secretion of the TFF2 factor and inhibit the secretion of the TNF-*α* factor in the GES-1 cell matrix after alcohol injury. TFF2 can form a gel barrier on the mucosal surface and inhibit further damage to the gastric mucosa from attacking factors, and increased TFF2 levels have been reported to accelerate the healing of gastric mucosal injury, while the expression of TNF-*α* can lead to inflammation [26,27]. Thus, the results showed that FCPP aqueous extract could protect and repair GES-1 cells by promoting the healing of gastric epithelial cells and inhibiting the pro-inflammatory factor TNF-*α*.

### 3.2. Effects of FCPP Aqueous Extract on Organ Index

Throughout the experimental trial, the behavior of rats was normal, and no toxic effects were observed in them upon consumption of the experimental diets (tested samples). The organ indexes of rats are illustrated in Table 2. In terms of thymus, there was a significant difference between the low-dose FCPP aqueous extract group and the normal control group (*p* < 0.05), and a significant difference was also found between the high-dose FCPP aqueous extract group and the vehicle group (*p* < 0.05). In the liver, there was statistical significance between the FCPP aqueous extract medium-dose group and the normal control group (*p* < 0.05). These differences might be due to inflammatory responses and weight errors. In other respects, there were no significant differences in the indexes of the thymus, liver, kidney and spleen between the groups with different doses of FCPP aqueous extract, the normal control group and the vehicle group (*p* > 0.05).The results indicate that the acute model had no significant effect on the organ indexes of rats in a short time.

### 3.3. Gastric Ulcer Evaluation and Histopathological Analysis

#### 3.3.1. Effects of FCPP Aqueous Extract on UI and UIR in Rats

**Figure 2:**
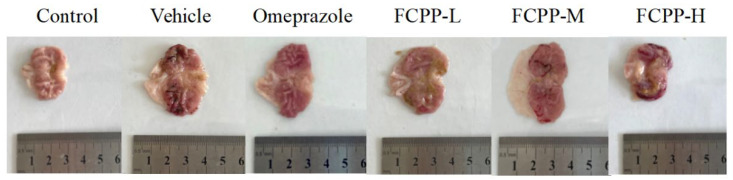
Photos of gastric tissue of rats in each group after incision along the great bend of cardia. Control: normal saline (i. g). Vehicle: normal saline (i. g). Omeprazole: omeprazole 50 mg·kg^−1^·d^−1^ (i. g). FCPP-L: FCPP aqueous extract 100 mg·kg^−1^·d^−1^ (i. g). FCPP-M: FCPP aqueous extract 400 mg·kg^−1^·d^−1^ (i. g). FCPP-H: FCPP aqueous extract 800 mg·kg^−1^·d^−1^ (i. g). The same below.

**Figure 3:**
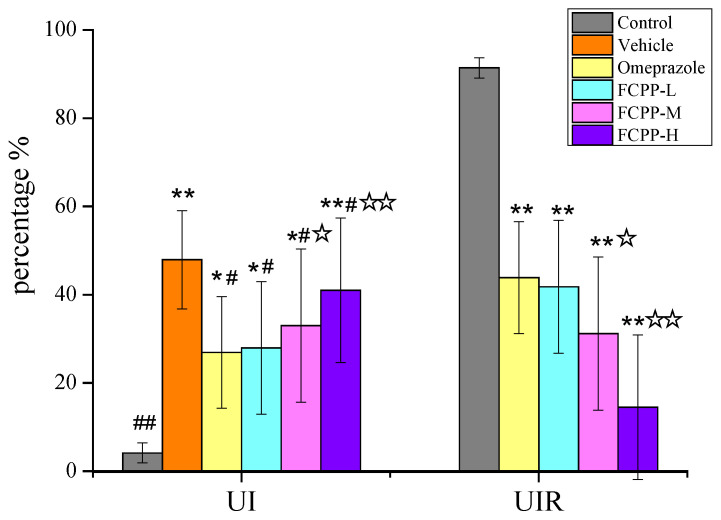
Gastric ulcer index and ulcer inhibition rate of rats in each group. Control: normal group. Vehicle: model group. Omeprazole: omeprazole group. FCPP-L: FCPP low-dose aqueous extract group. FCPP-M: FCPP medium-dose aqueous extract group. FCPP-H: FCPP high-dose aqueous extract group. * *p* < 0.05, ** *p* < 0.01 versus control group; ^#^ *p* < 0.05, ^##^ *p* < 0.01 versus the vehicle group; ^☆^ *p* < 0.05, ^☆☆^ *p* < 0.01 the low, medium and high FCPP extract doses versus the omeprazole-treated group, *n* = 10. The same below.

To determine the protective effect of FCPP aqueous extract on gastric tissues after alcohol damage by macroscopically observing the damage status of rat gastric tissues and calculating the UI and UIR indices. The macroscopic morphology of the stomach tissues of rats in the control group was normal without obvious hemorrhagic lesions (Figure 2), and its UI was only 4.13%, while the UIR high at 91.39% (Figure 3). In the vehicle group, hemorrhagic lesion was obvious, and the ulcer was the most serious, with the UI reaching 47.93%. The UI in the omeprazole group was 26.91%, which was significantly lower than that of the model group (*p* < 0.05), and the UIR in the omeprazole group was 43.85%. Compared with the model group and the omeprazole group, the ulcer lesions in the gastric tissues of rats treated with FCPP aqueous extract were significantly reduced. Among them, the UI of the medium-dose and high-dose groups of FCPP aqueous extract were 32.99% and 40.98%, respectively, which were significantly lower than that of the model group (*p* < 0.05), and the UIR were 31.17% and 14.50%, respectively. The low-dose FCPP aqueous extract group had the best prevention effect. Its gastric UI was 27.91%, which was significantly lower than that of the model group (*p* < 0.05), and the UIR was 42.78%, which was not significantly different from the positive control group (i.e., the omeprazole group) (*p* > 0.05).

#### 3.3.2. Histopathological Analysis

**Figure 4:**
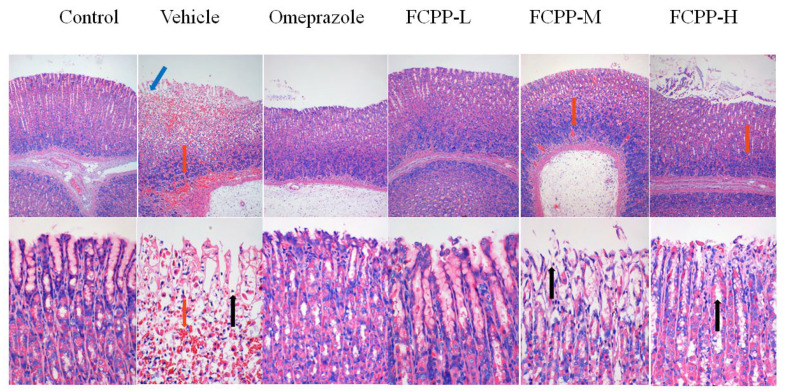
Morphological structure of gastric tissue in each group (HE staining, 100- and 400-times magnification in the upper and lower rows, respectively). Control: normal group. Vehicle: model group. Omeprazole: omeprazole group. FCPP-L: FCPP aqueous extract low-dose group. FCPP-M: FCPP aqueous extract medium-dose group. FCPP-H: FCPP aqueous extract high-dose group. The red arrow indicates local bleeding, the black arrow indicates inflammatory cell infiltration and the blue arrow indicates glandular gastric mucosal cell necrosis.

The H&E staining section of the gastric tissue structure of rats is shown in Figure 4. In the normal control group, the epithelial gland cells of gastric mucosa were neatly arranged with a complete structure and distinct layers, and without bleeding or inflammatory cell infiltration. In the vehicle group, there was severe exfoliation of mucosal epithelial glands, and the structures of the mucosal layer, the submucosal layer and the muscle layer were disordered, accompanied by the appearance of abnormal tissue structures (such as local bleeding and infiltration of inflammatory cells). However, these pathological signals were significantly relieved with the treatment of FCPP aqueous extract. In the low-dose FCPP aqueous extract group, the structure of the gastric glands in rats was relatively orderly, and no edema and inflammatory cell infiltration were observed in the submucosa. Furthermore, the gastric tissue structure in the low-dose group was the closest to that of normal rats. Although the submucosal edema was effectively relieved in the medium- and high-dose FCPP aqueous extract groups, there were still local abnormal tissue structures, such as scattered bleeding and inflammatory cell infiltration. The results demonstrated that the aqueous extract of FCPP had a protective effect on the gastric mucosa of rats with alcohol-induced acute gastric ulcers.

### 3.4. Effects of FCPP Aqueous Extract on the Level of SOD and MDA in Rats

**Figure 5:**
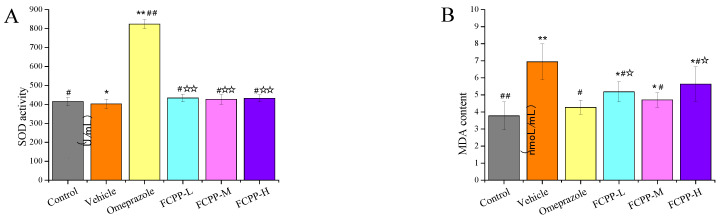
(**A**) SOD activity in serum of rats in each group. (**B**) MDA contents in serum of rats in each group. Control: normal group. Vehicle: model group. Omeprazole: omeprazole group. FCPP-L: FCPP low-dose aqueous extract group. FCPP-M: FCPP medium-dose aqueous extract group. FCPP-H: FCPP high-dose aqueous extract group. * *p* < 0.05, ** *p* < 0.01 versus control group; ^#^ *p* < 0.05, ^##^ *p* < 0.01 versus the vehicle group; ^☆^ *p* < 0.05, ^☆☆^ *p* < 0.01 the low, medium and high FCPP extract doses versus the omeprazole-treated group, *n* = 10.

Oxidative stress also occurs in the pathological process of gastric ulcer. Lipid peroxidation is a stress response induced by oxidation, and is an important pathogenesis of gastric mucosal injury [28]. SOD and MDA are potentially important indexes of antioxidant capacity in vivo, which can directly reflect the rate and intensity of lipid peroxidation and indirectly reflect the degree of tissue peroxidation damage [29]. The results of SOD activity and MDA contents in the serum of rats are illustrated in Figure 5A. The SOD activity (402 U/mL) in the vehicle group was the lowest, and the highest content of SOD appeared in the positive control group (823.58 U/mL). There was no significant difference in the SOD activity among the FCPP aqueous extract treatment groups (*p* > 0.05). Among these, the SOD activity of the low-dose group was 433.73 U/mL, which was significantly higher than that of the vehicle group under the same dose of alcohol damage (*p* < 0.05), indicating that the treatment of FCPP aqueous extract at a low dose could improve the SOD activity and enhance the antioxidant capacity of the body. In the MDA results (Figure 5B), the lowest MDA content was 3.77 mol/mL in the normal control group, and the highest MDA content was 6.94 mol/mL in the vehicle group. In the FCPP aqueous extract treatment groups, the lowest MDA content (4.1 mol/mL) appeared in the medium-dose group, which was significantly lower than that of the model group (*p* < 0.05) under the same alcohol injury conditions. The results demonstrated that the treatment of FCPP aqueous extract at medium dose could reduce the MDA value in serum and prevent oxidation of the body.

### 3.5. Effects of FCPP Aqueous Extract on the Expression of Pro-Inflammatory and Anti-Inflammatory Factors

**Figure 6:**
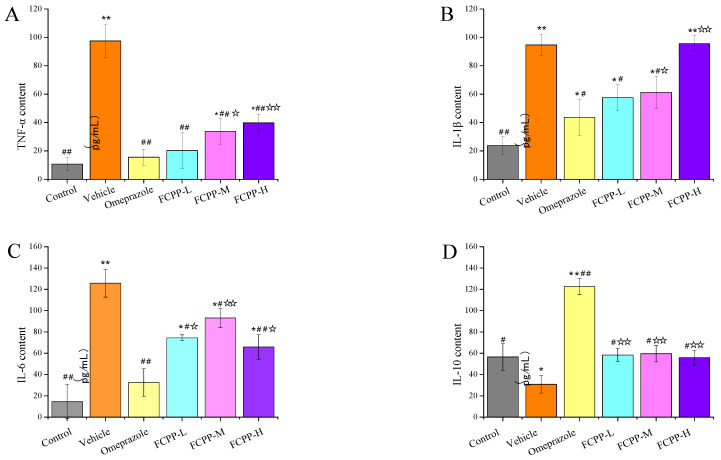
The contents of TNF-*α* (**A**), IL-1*β* (**B**), IL-6 (**C**) and IL-10 (**D**) in serum of rats in each group. Control: normal group. Vehicle: model group. Omeprazole: omeprazole group. FCPP-L: FCPP low-dose aqueous extract group. FCPP-M: FCPP medium-dose aqueous extract group. FCPP-H: FCPP high-dose aqueous extract group. * *p* < 0.05, ** *p* < 0.01 versus the control group; ^#^ *p* < 0.05, ^##^ *p* < 0.01 versus the vehicle group; ^☆^ *p* < 0.05, ^☆☆^ *p* < 0.01 the low, medium and high FCPP extract doses versus the omeprazole-treated group, *n* = 10.

Tumor necrosis factor-*α* (TNF-*α*) and interleukin family (IL) are two key inflammatory cytokines, which are closely related to gastric mucosa injury. Once the gastric mucosa is damaged, the inflammatory response process will be activated [30,31]. In this study, the levels of TNF-*α*, IL-1*β*, IL-6 and IL-10 in rat serum were determined. TNF-*α*, IL-1*β* and IL-6 are all pro-inflammatory cytokines, while IL-10 is an anti-inflammatory cytokine, which can inhibit the production of inflammatory cytokines by activating macrophages [32]. As shown in Figure 6, the content (97.5 pg/mL) of TNF-*α* in the vehicle group was significantly higher than that of the other groups (*p* < 0.01), and the lowest content of TNF-*α* existed in the control group and the positive control group, which was 12.75 pg/mL and 15.58 pg/mL, respectively: extremely significantly lower than that of the vehicle group (*p* < 0.01). Among the FCPP aqueous extract treatment groups, the lowest content of TNF-*α* was 20.25 pg/mL in the low-dose group, and its content showed an upward trend with the increase of the dose of FCPP aqueous extract. The IL-1*β* level in the FCPP-H group showed no significant difference from the vehicle group (*p* > 0.05), which was the highest among other groups, and the lowest value of IL-1*β* appeared in the normal control groups. Among the FCPP aqueous extract treatment groups, the content of IL-1*β* in the low-dose group was the lowest (57.57 pg/mL), which was drastically lower than that of the vehicle group (*p* < 0.05), and also showed an increasing trend with the increase of the dose of FCPP aqueous extract. In terms of IL-6 content, there were significant differences (*p* < 0.05) between the sample groups. With the increase of FCPP aqueous extract dose, the IL-6 content showed an upward trend, followed by a decline. Among them, the high-dose group of FCPP aqueous extract could significantly reduce the level of IL-6 (65.91 pg/mL), which was evidently lower than that of the vehicle group (125 pg/mL) (*p* < 0.01). For anti-inflammatory cytokine IL-10, it could be seen that the anti-inflammatory effect of the positive control group was the best, with IL-10 content up to 122 pg/mL, which was significantly higher than that of the other groups (*p* < 0.01). The IL-10 content of the medium-dose FCPP aqueous extract group ranked second, with a value of 59.50 pg/mL, which was significantly higher than that of the vehicle group (*p* < 0.05). The low-dose FCPP aqueous extract group had an IL-10 content of 58.33 pg/mL, which was also significantly higher than that that of the vehicle group (*p* < 0.05). In conclusion, the results showed that FCPP aqueous extract could effectively inhibit the increase of cytokines TNF-*α*, IL-1*β* and IL-6 in the serum of rats, and had a promoting effect on the increase of anti-inflammatory cytokine IL-10.

### 3.6. Effects of FCPP Aqueous Extract on NF-*κ*B and NLRP3 Anti-Inflammatory Pathways after Ethanol-Induced Gastric Mucosal Injury

**Figure 7:**
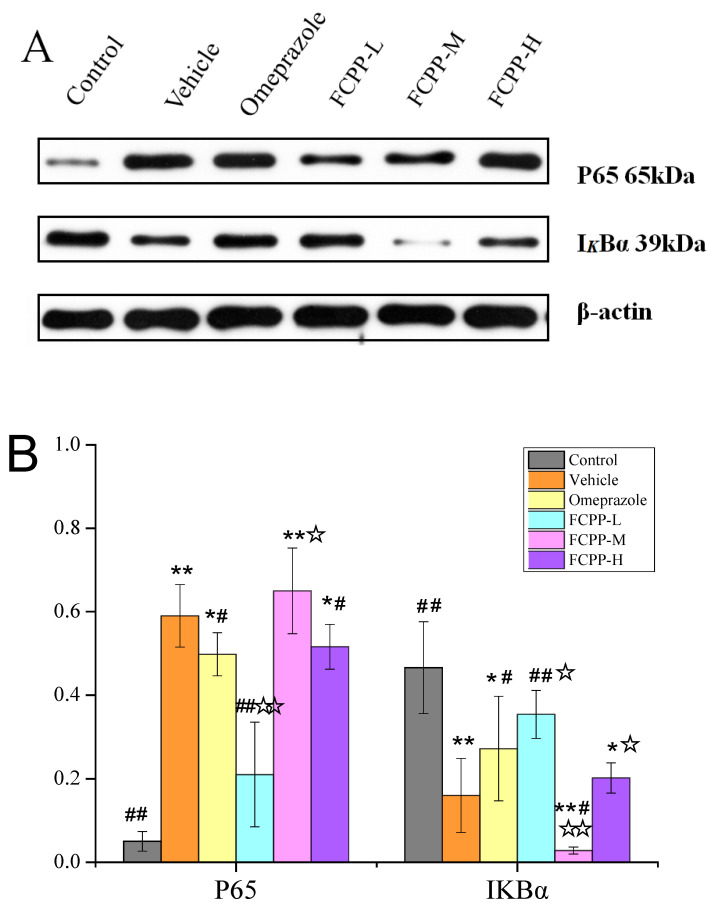
(**A**) Effect of FCPP aqueous extract on protein expression levels of P65 and IκB-*α* in the gastric tissues of rats. (**B**) Expression of P65 and I*κ*B*α* in the NF-*κ*B pathway in the gastric tissue of rats in each group. Control: normal group. Vehicle: model group. Omeprazole: omeprazole group. FCPP-L: FCPP low-dose aqueous extract group. FCPP-M: FCPP medium-dose aqueous extract group. FCPP-H: FCPP high-dose aqueous extract group. * *p* < 0.05, ** *p* < 0.01 versus the control group; ^#^ *p* < 0.05, ^##^ *p* < 0.01 versus the vehicle group; ^☆^ *p* < 0.05, ^☆☆^ *p* < 0.01 the low, medium and high FCPP extract doses versus the omeprazole-treated group, *n* = 3.

The activation of the NF-*κ*B/NLRP3 signaling pathway is an important link in the inflammatory response [33]. Under non-stress conditions, the trimer formed by the binding of NF-*κ*B protein and its inhibitory protein I*κ*B*α* was stably free in the cytoplasmic matrix, and prevented the NF-*κ*B protein from entering the nucleus. Once stimulated by inflammatory signals, I*κ*B*α* will be phosphorylated or degraded by kinase, and NF-*κ*B will be activated and released, and free to bind to target genes in the nucleus, resulting in inflammation [34]. As depicted in Figure 7A,B, the P65 protein expression of NF-*κ*B increased significantly in the vehicle group compared with the normal control group (*p* < 0.01), while the expression of I*κ*B*α* decreased significantly (*p* < 0.01). These results indicated that part of I*κ*B*α* was phosphorylated in the vehicle group, and NF-*κ*B was activated to enter the nucleus and bind with related genes, which promoted the occurrence of inflammation and increased the degree of gastric ulcers. The expression of the P65 protein decreased significantly in rats treated with omeprazole positive drugs (*p* < 0.05), and the expression of I*κ*B*α* was significantly higher than that in the vehicle group (*p* < 0.05).Among the FCPP water extract treatment groups, the low-dose group could inhibit the expression of P65 protein and promote the expression of the I*κ*B*α* protein with a value of 21% and 35%, which was 60% and 57% higher than those of the vehicle group, respectively (*p* < 0.01). These results demonstrated that FCPP aqueous extract at a certain dose could inhibit the activation of the NF-*κ*B pathway by inhibiting the phosphorylation of the I*κ*B*α* protein, thereby restraining the occurrence of inflammation and preventing a gastric ulcer. The inhibitory effects of medium and high doses of FCPP aqueous extract were significantly lower than those of the low dose (*p* < 0.05).

**Figure 8:**
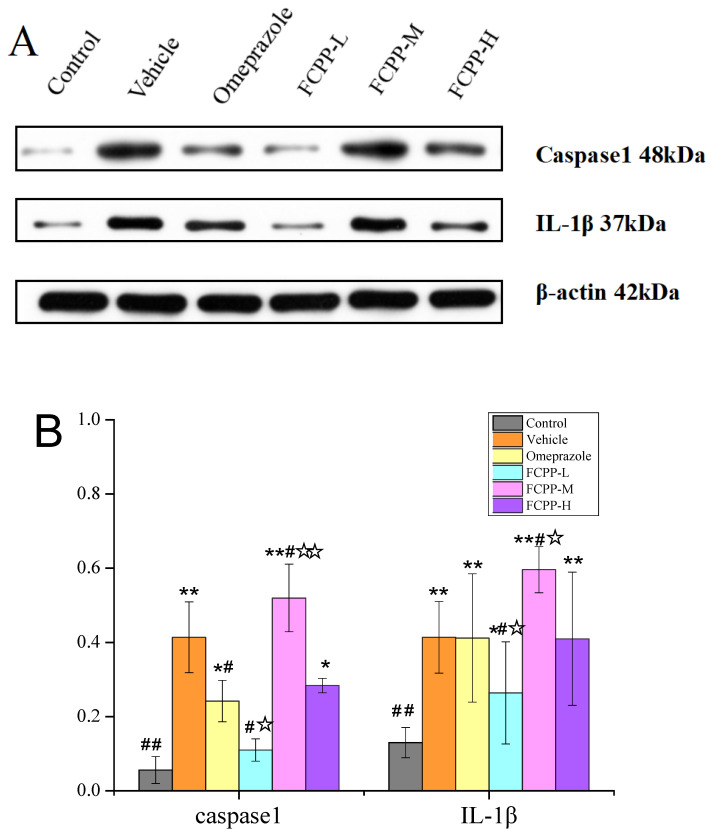
(**A**) Effect of FCPP aqueous extract on protein expression levels of caspase-1 and IL-*β* in the gastric tissues of rats. (**B**) Expression of caspase1 and IL-1*β* in the NLRP3 pathway in the gastric tissues of rats in each group. Control: normal group. Vehicle: model group. Omeprazole: omeprazole group. FCPP-L: FCP Plow-dose aqueous extract group. FCPP-M: FCPP medium-dose aqueous extract group. FCPP-H: FCPP high-dose aqueous extract group. * *p* < 0.05, ** *p* < 0.01 versus the control group; ^#^ *p* < 0.05, ^##^ *p* < 0.01 versus the vehicle group; ^☆^ *p* < 0.05, ^☆☆^ *p* < 0.01 the low, medium and high FCPP extract doses versus the omeprazole-treated group, *n* = 3.

Activated caspase-1 protein is a key transduction signal in the inflammatory response, which further activates the precursor of IL-1*β* and causes the activated IL-1*β* to be secreted extracellular. The two proteins participate in the inflammatory response by generating an immune response in the body. The activation of caspase-1 promotes IL-1*β* secretion [35]. Compared with the normal control group, the expression of caspase-1 protein (41%) and IL-1*β* protein (41%) in the vehicle group increased significantly, as shown in Figure 8A,B (*p* < 0.01), which further indicated that alcohol could cause inflammation and increase the degree of gastric ulcers in the vehicle group. Gavage of omeprazole could inhibit the expression of caspase-1 protein and IL-1*β* protein to a certain extent, thus preventing inflammation [36]. Among the FCPP aqueous extract groups, the expressions of the two proteins in the FCPP-L group declined dramatically (*p* < 0.05)—11% and 26% lower than those of the vehicle group, respectively. In the FCPP-M group, the content of caspase-1 and IL-1*β* protein were 52% and 60%, respectively, and the equivalent values for the FCPP-H group were 28% and 41%, respectively. The inhibition effect was significantly lower than that of the low-dose group (*p* < 0.05). These results indicated that FCPP aqueous extract at a certain dose could inhibit inflammation and prevent gastric ulcer by regulating the expression of caspase-1 protein and IL-1*β* protein.

### 3.7. Analysis of the Major Active Components of FCPP Aqueous Extract

**Figure 9:**
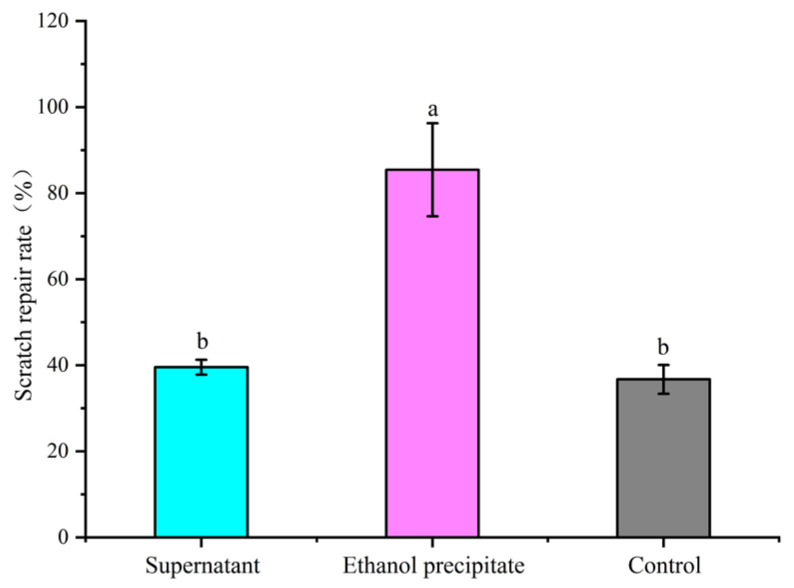
Effects of the ethanol precipitate (deproteinated) and supernatant of FCPP aqueous extract on GES-1 scratch repair rate. Control: cultured with cell culture medium only. Supernatant: cultured with cell culture medium containing 100 µg/mL supernatant of FCPP aqueous extract after scratch injury. Ethanol precipitate: cultured with cell culture medium containing 100 µg/mL ethanol precipitate of FCPP aqueous extractafter scratch injury, *n* = 3. Different letters indicate significant differences (*p* < 0.05), and the same letters indicate nonsignificant differences (*p* > 0.05).

The ability of GES-1 cells to proliferate and migrate is an important factor affecting the healing of mucosal injury, and promoting cell proliferation and migration to the site of mucosal injury is the key to improving the quality of mucosal protection [37]. Cell scratch assay was often used to evaluate the migration ability of GES-1 cells. As depicted in Figure 9, the scratch repair rate of the ethanol precipitate of FCPP aqueous extract showed a significant increase compared to both the supernatant of FCPP aqueous extract and the control group (*p* < 0.05), and increased by 115.95% and 132.64%, respectively, indicating that it was mainly the macromolecular components in the FCPP aqueous extract that had a repair role in scratched GES-1 cells. The chemical composition analysis of the ethanol precipitate found that it contained 70% sugar, 0.42% protein, 0.04% polyphenols and 40.9% uronic acid, as shown in Table 3. Ethanol precipitation in hot water is generally the first step to extract crude polysaccharides [21], so it was speculated that the polysaccharides in the FCPP aqueous extract might play a major role in the gastroprotective effects of the FCPP aqueous extract.

## 4. Discussion

Gastric ulcers can be caused by a variety of factors, such as alcohol, Helicobacter pylori, non-steroidal anti-inflammatory drugs and stress [38]. Alcohol is one of the typical aggressive factors. The inhibition of cell proliferation, inflammatory cell infiltration, oxygen free radical reduction of lipid peroxidation and protein oxidation and other processes are all involved in the pathogenesis of ethanol-induced gastric mucosa injury [39]. In vitro cell experiments on the protective mechanism of FCPP on gastric mucosa showed that an FCPP aqueous extract could promote the secretion of TFF2 and inhibit the secretion of TNF-*α* after the cells were damaged by alcohol, and thus play a protective and reparative role on GES-1 cells. By inhibiting the expression of intercellular adhesion molecules, TFF2 can change cell connections and induce normal epithelial cells to migrate to the damaged area to replace the damaged epithelial cells [40,41]. The in vivo animal experiment showed that, after the intervention of FCPP aqueous extract in experimental SD rats, the gastric ulcer index of rats in the low-dose group was only 27.91, which was significantly lower than that of the model group (*p* < 0.01). While the ulcer inhibition rate was as high as 42.78, and there were no significant differences between them and those in the positive control group (*p* > 0.05). This indicated that, when high concentrations of alcohol attacked the stomach, FCPP could effectively protect the integrity of the gastric mucosa structure and achieve the effect of preventing gastric ulcers. The results were similar to those of the Vitex doniana crude extract and water extract of American ginseng in preventing gastric ulcer [42,43]. When the body is stimulated by alcohol, protein oxidation reaction and lipid peroxidation will be triggered, leading to the production of a large number of free radicals and a decrease in the body’s antioxidant level [44,45]. Therefore, alcohol-related acute gastric ulcer can also be prevented by improving the body’s peroxidation stress state. At present, most of the drugs for the treatment of gastric ulcer on the market are only for the initial treatment of gastric ulcer by simply reducing the level of gastric acid, but this method cannot suppress oxidation, which is an important factor influencing the long course of disease. Furthermore, gastric ulcers are difficult to cure and prone to relapse when treated by Western drugs [46]. The results demonstrated that FCPP aqueous extract could improve SOD activity and antioxidant capacity, and there was no significant difference among all groups of FCPP aqueous extract (*p* > 0.05). However, the medium dose of FCPP aqueous extract could significantly reduce the MDA value (*p* < 0.05), exhibiting the effect of preventing oxidative damage. The occurrence of gastric ulcer is closely related to inflammatory response. After the gastric mucosa is stimulated, the inflammatory signaling pathway is further activated to promote the secretion of inflammatory factors and inhibitory growth factors, and the balance of gastric mucosal defense system will then be destroyed [47]. The NF-*κ*B/NLRP3 inflammasome pathway is an important pathway for regulating the expression and secretion of inflammatory cytokines, which can promote the secretion of inflammatory cytokines TNF-*α*, 1L-6 and 1L-1*β* and inhibit the secretion of anti-inflammatory cytokine IL-10 [48,49,50]. In this study, FCPP aqueous extract could effectively inhibit the increase of TNF-*α*, IL-1*β* and IL-6 contents in rat serum, and promote the increase of IL-10 content to a certain extent. The activation of the NLRP3 inflammasome is regulated by NF-*κ*B. When exposed to extracellular stimulation, pro-caspase-1 is cleaved to activate caspase-1, then IL-1*β* and IL-18 precursors are cleaved to IL-1*β* and IL-18, which release in vitro [51]. FCPP aqueous extract at low dose (100 mg/kg) exhibited the highest efficacy for inhibiting the expression of the P65 protein and promoting the expression of I*κ*B*α* protein, which was 60% and 57% higher than those of the vehicle group, respectively (*p* < 0.01). Furthermore, the low dose of FCPP exhibited the highest efficacy for downregulating the protein expression of caspase 1 and IL-1 beta; the expression of caspase-1 protein and IL-1*β* protein was reduced by 73% and 37% compared with those of the model group (*p* < 0.05). FCPP aqueous extract might prevent gastric ulcers by inhibiting the phosphorylation of I*κ*B*α* and the activation of the NF-*κ*B pathway, thereby further regulating the decrease of the expression levels of caspase-1 and IL-1*β*, and inhibiting inflammation. The low dose of FCPP exhibited the highest anti-inflammatory effect [52]. FCPP aqueous extract demonstrated a stronger protective effect on the gastric mucosa at medium (400 mg/kg) and low doses compared to high dose (800 mg/kg), potentially due to an excessive intake of FCPP aqueous extract producing toxic and side effects. In our study, we examined the activities/levels of SOD, MDA, TNF-*α*, IL-1*β*, IL-6 and IL-10 in serum, which are produced and transported into the blood by various tissues of the body, and can reflect the antioxidant and anti-inflammatory effects of rats. In conclusion, FCPP aqueous extract had certain protective effects on the gastric mucosa of rats after alcohol injury, and the potential mechanism was to protect the gastric mucosa by improving the body’s antioxidant stress ability, and regulating the secretion of inflammatory factors via activating the NF-*κ*B and NLRP3 pathways. Many studies have also shown that polysaccharides have better gastroprotective activity [12,29,47], and, in our subsequent experimental cell and chemical composition analysis, we also initially found that the main chemical that exerts gastroprotective activity in the aqueous extract may be polysaccharides, thus our subsequent experiments will also focus on polysaccharides for their structures.

## 5. Conclusions

In this study, the protective effect of the aqueous extract of FCPP on gastric mucosa in rats with alcohol-induced gastric injury was studied in both in vitro and in vivo experiments. In vitro studies showed that FCPP aqueous extract could promote the secretion of TFF2 and inhibit the secretion of TNF-*α* in GES-1 cells damaged by alcohol, playing a protective and reparative role in the cells. In vivo studies indicated that FCPP aqueous extract had a good protective effect on the gastric stomach of rats, which was evidenced by the significant decrease of the ulcer index of gastric tissue of the rats treated with FCPP aqueous extract. FCPP aqueous extract could also increase the SOD activity and enhance the antioxidant capacity of rats. Furthermore, FCPP aqueous extract could significantly reduce the MDA value in serum of rats to prevent oxidative damage. The aqueous extract of FCPP could effectively inhibit the increase of cytokines TNF-*α*, IL-1*β* and IL-6 in the serum of rats, and promote the increase of anti-inflammatory cytokine IL-10 to some extent. FCPP aqueous extract at low dose (100 mg/kg) could inhibit the expression of P65 protein and promote the expression of I*κ*B*α* protein in the gastric tissue of rats, which were 60% and 57% higher than those of the vehicle group, respectively (*p* < 0.01). Moreover, the expression of caspase-1 protein and IL-1*β* protein in the gastric tissue of rats treated with FCPP aqueous extract was reduced by 73% and 37%, respectively, compared with those of the model group, indicating that the aqueous extract of FCPP could protect gastric mucosa by inhibiting inflammation. In conclusion, this study demonstrated that the aqueous extract of FCPP has a good protective effect on gastric mucosa. A GES-1 cell scratch assay showed that the polysaccharides in FCPP aqueous extract might be the main components that exerted gastroprotective activity. The structure of FCPP polysaccharide can be further studied to explore the structure–activity relationship of its gastroprotective activity.

## Figures and Tables

**Table 1:** Animal Group and Drug Administration Scale. FCPP: finger citron pickled products. Control: normal group; Vehicle: model group; Omeprazole: omeprazole group; FCPP-L: FCPP aqueous extract low-dose group; FCPP-M: FCPP aqueous extract medium-dose group; FCPP-H: FCPP aqueous extract high-dose group.

Experimental Groups	Number of Animals	Dose of Administration	Modeling
Control	10	normal saline (5 mL·kg^−1^·d^−1^)	normal saline (5 mL·kg^−1^)
Vehicle	10	normal saline (5 mL·kg^−1^·d^−1^)	anhydrous ethanol (5 mL·kg^−1^)
FCPP-H	10	FCPP aqueous extract (800 mg·kg^−1^·d^−1^, 5 mL·kg^−1^·d^−1^)	anhydrous ethanol (5 mL·kg^−1^)
FCPP-M	10	FCPP aqueous extract (400 mg·kg^−1^·d^−1^, 5 mL·kg^−1^·d^−1^)	anhydrous ethanol (5 mL·kg^−1^)
FCPP-L	10	FCPP aqueous extract (100 mg·kg^−1^·d^−1^, 5 mL·kg^−1^·d^−1^)	anhydrous ethanol (5 mL·kg^−1^)
Omeprazole	10	Omeprazole (50 mg·kg^−1^·d^−1^, 5 mL·kg^−1^·d^−1^)	anhydrous ethanol (5 mL·kg^−1^)

**Table 2:** Comparison of organ index in rats. Control: normal group. Vehicle: model group. Omeprazole: omeprazole group. FCPP-L: FCP low-dose aqueous extract group. FCPP-M: FCPP medium-dose aqueous extract group. FCPP-H: FCPP high-dose aqueous extract group. * *p* < 0.05, versus the normal control group; ^#^ *p* < 0.05, versus the vehicle group, *n* = 10.

Organ Index (mg/g)				
Group	Thymus	Spleen	Kidney	Liver
Control	2.39 ± 0.3	3.23 ± 0.49	8.13 ± 0.65	31.03 ± 3.02
Vehicle	2.48 ± 0.52	3.06 ± 0.28	8.17 ± 0.76	33.52 ± 4.28
Omeprazole	2.43 ± 0.26	3.1 ± 0.37	8.13 ± 0.38	33.83 ± 1.82
FCPP-L 100 mg/kg	2.6 ± 0.25 *	3.14 ± 0.29	8.19 ± 0.6	33.48 ± 2.48
FCPP-M 400 mg/kg	2.37 ± 0.34	3.17 ± 0.4	8.23 ± 0.5	34.24 ± 1.64 *
FCPP-H 800 mg/kg	2.28 ± 0.28 ^#^	3.02 ± 0.46	8.07 ± 0.42	32.58 ± 1.53

**Table 3:** Chemical compositions of the ethanol precipitate (deproteinated) of FCPP aqueous extract.

Sugar Content (%)	Protein Content (%)	TPC ^a^ (%)	UAC ^b^ (%)
70 ± 0.001	0.420 ± 0.086	0.04 ± 0.01	40.90 ± 1.88

^a^: TPC: total phenolic content; ^b^: UAC: uronic acid content.

## Data Availability

Data is contained within the article.
